# Comparison of ATR–FTIR and O-PTIR Imaging Techniques for the Characterisation of Zinc-Type Degradation Products in a Paint Cross-Section

**DOI:** 10.3390/molecules27196301

**Published:** 2022-09-24

**Authors:** Lynn Chua, Agnieszka Banas, Krzysztof Banas

**Affiliations:** 1 Heritage Conservation Centre, National Heritage Board, Singapore 619104, Singapore; 2 Singapore Synchrotron Light Source, National University of Singapore, Singapore 117603, Singapore

**Keywords:** O-PTIR, ATR–FTIR imaging, paint cross-section, zinc lactate, gordaite, zinc soap, conservation

## Abstract

ATR–FTIR (attenuated total reflection–Fourier-transform infrared) microscopy with imaging is widely used in the heritage field to characterise complex compositions of paint cross-sections. However, some limitations include the need for ATR crystal contact with the sample and the inability to resolve particle size below the IR diffraction limit. Recently, a novel O-PTIR (optical-photothermal infrared) spectroscopy technique claimed to open a new avenue for non-invasive, efficient, and reliable analysis at sub-micron resolution. O-PTIR produces transmission-like FTIR spectra for interpretation, without the need to touch the sample, which are highly favourable attributes for analysing heritage samples. This paper reports the comparison of O-PTIR and ATR–FTIR techniques applied to a cross-section embedding a thin paint fragment that delaminated from a late 19th to early 20th-century oil portrait. The hazy paint fragment consisted of zinc soaps (both crystalline and amorphous), gordaite (NaZn_4_Cl(OH)_6_SO_4_·6H_2_O), and zinc lactate, that could not all be well-resolved with ATR–FTIR imaging. With O-PTIR analysis, the degradation compounds could be resolved at sub-micron resolution with an equivalent or better signal-to-noise ratio. This case study shows how the two techniques can be used to obtain comprehensive information at a broad level with ATR–FTIR and a detailed level with O-PTIR.

## 1. Introduction

### 1.1. ATR–FTIR

In cultural heritage, it is often desirable to analyse the chemical make-up of the paint layers and stratigraphies to determine the artist’s technique, choice of materials, and condition of the paints to inform conservation work. As a result, many papers describing the usage of ATR–FTIR (attenuated total reflection–Fourier-transform infrared), chemical imaging, or mapping to analyse paint samples were developed for these purposes [1,2,3]. It is very important to notice that paint samples coming from heritage works of art are very demanding to study for the following reasons: the cross-sections consist of heterogeneous multi-layers (e.g., pigment, filler, binder, resin), they are usually small in size (sampling from artworks is limited), they are unknown (no prior information of the material used, no standard formula or recipe), and likely underwent chemical changes from deterioration and ageing processes. When analysing such complex paint samples, FTIR imaging is particularly useful, especially when samples contain layers of interest that are too thin for discrete micro-sampling or adjacent layers containing different compounds are close in colour and not visually discernible.

Researchers typically select an ATR objective mounted to the IR microscope if the samples to analyse are neither transparent nor reflective-like, as in the case of paint cross-sections. Unfortunately, ATR experiments require intimate contact between the analysed sample and the ATR crystal, which may lead to sample deformation and even loss of precious information that cannot be obtained again with re-polishing. Nevertheless, this contact is needed as only in this way can evanescent waves penetrate the sample to the depth of 0.3–3 microns, depending on the angle of incidence, wavelength, and refractive indices of the ATR crystal and sample. Additionally, germanium is typically selected for ATR crystals in paint analysis for its high refractive index and better spatial resolution. However, it can be easily scratched or even broken over usage, leading to the need for a costly replacement. During experiments, particular care must be taken to clean the crystal after each measurement to avoid cross-contamination.

The ATR objective coupled to an IR microscope equipped with a multi-channel detector focal plane array (FPA) enables the collection of a 2D map with spectra corresponding to the number of detector elements during one measurement. This method is already established in chemistry, biology, and the analysis of paint samples [1,2,3]. If a 64 × 64 FPA detector is used with a 20X ATR objective, 4096 spectra are simultaneously recorded, but the amount of light absorbed from a very small area of 1.1 × 1.1 µm^2^ leads to rather noisy spectra. The pixel size given here should not be treated as the real spatial resolution, which is limited by diffraction to about one wavelength (the Rayleigh criterion); thus, features smaller than a few micrometres cannot be resolved.

### 1.2. O-PTIR

Recently, a novel O-PTIR spectromicroscopy technique claimed to break the diffraction limit of traditional IR spectroscopy. In O-PTIR, a pulsed, tunable IR laser is used to illuminate the sample. When a selected wavenumber is absorbed within the sample, the photothermal effect occurs, which is detected by the visible probe. That is why the spatial resolution is independent of the IR light, and sub-micron resolution is achieved. The experiment is performed in non-contact mode, requires little or no sample preparation, and leaves the sample in pristine condition after analysis. The same sample can be analysed by other analytical methods for a complementary study. O-PTIR measurements, similar to FTIR spectroscopy, provide information about absorption bands collected as a function of wavenumber [cm^−1^]. Even if O-PTIR measurements are performed in a non-contact reflection mode, the quality of the obtained spectra is comparable to FTIR transmission-like infrared spectra and they are artefact-free [4].

The mIRage IR microscope, which utilises the O-PTIR technique, is a unique system commercialised by the Photothermal Spectroscopy Corp (PSC) in 2017. The mIRage operates in an easy-to-use far-field mode and offers significantly higher sensitivity than FTIR spectromicroscopy. Within a few years, this instrument has been used in a wide range of applications, including investigation of polymeric and non-polymeric materials, life science and complex pharmaceutical samples, and micro-electronics contamination.

In the field of cultural heritage, O-PTIR spectroscopy is considered relatively new. It has only been applied to a few cases: (1) characterisation of geranium lake pigment in a polished cross-section from Van Gogh’s painting [5], (2) direct characterisation of corrosion products on 16th-century glass and decorative brass elements without sampling [6], and (3) characterisation of zinc soaps and pigments in a historic oil paint cross-section [7]. Thus far, the positive results advocate for O-PTIR as a non-invasive, sub-micron-resolution imaging tool that can be potentially applied to other intricate samples in the heritage field.

In this study, we compare the novel O-PTIR and the classical ATR–FTIR imaging techniques on the same paint cross-section sample obtained from a late 19th to early 20th-century oil painting. The hazy paint fragment delaminated from the surface of the painting was embedded in resin and polished prior to analysis. A key advantage proposed for the O-PTIR technique, and our primary interest for the comparative study, is its ability to accurately characterise and spatially resolve different compounds below the IR diffraction limit. The paint sample (about 15 µm thickness) containing different types of degradation compounds with granularity around a few microns makes it an ideal and realistic sample for this comparison study.

## 2. Methods and Materials

### 2.1. Sample Preparation and Microscopic Examination

**Figure 1:**
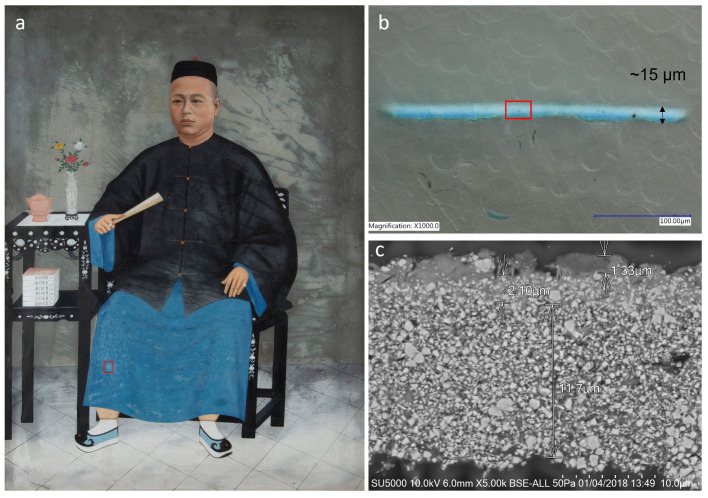
(**a**) *Portrait of Mr Tan Beng Wan*, Oil on canvas, Collection of The Peranakan Museum, National Heritage Board. Gift of Mr and Mrs Tan Choon Hoe. Red box denotes the location of the sample. Cross-section of the hazy sample in (**b**), reflected ring light microscopy with the red box denoting the scanning electron microscopy (SEM) image in (**c**).

A thin paint fragment (15 µm thickness) that had delaminated from a “hazy” oil painting—“*Portrait of Mr Tan Beng Wan*” (late 19th to early 20th century) (Figure 1a)—was made available for testing. The sample was embedded in *Clarocit* acrylic resin, ground, and polished to reveal the cross-section of interest. Dry polishing of the cross-section was achieved using the *MOPAS* polisher with successive grades of *Micromesh* abrasive cloths up to 12,000 grit. The sample was then examined with a Keyence digital microscope coupled with a VH-Z100 zoom lens and Hitachi SU5000 field-emission scanning electron microscope (FE-SEM) with a backscattering detector (BSD) at 50 Pa, using the BSE-ALL mode.

### 2.2. ATR–FTIR Spectroscopy

ATR–FTIR spectromicroscopy with chemical imaging was performed at the Heritage Conservation Centre (HCC), Singapore. The Agilent Cary 670 FTIR spectrometer and Cary 620 microscope system consists of a 15 X objective (NA = 0.62) and a 64 × 64 focal plane array (FPA) detector. With a micro-Ge (germanium) ATR inserted at the microscope objective, this configuration gave a field of view of about 70 × 70 µm (1.1 µm^2^ pixels) and the simultaneous acquisition of 4096 spectra (in seconds to minutes).

The sample was placed on a motorised stage with a micro-vice and gently brought into sufficient contact with the ATR crystal. The pressure applied to the sample was adjusted to optimise signal intensity. The spectra were collected between 4000 and 900 cm^−1^ with the co-addition of 64 scans at 4 cm^−1^ resolution. FTIR chemical images of the sample were obtained by selecting the integrated absorbance of the characteristic wavenumbers of interest.

### 2.3. O-PTIR Spectroscopy

O-PTIR experiments were performed at the branch of the upgraded ISMI beamline (at the Singapore Synchrotron Light Source) using a mIRage microscope manufactured by the Photothermal Spectroscopy Corp. (PSC), Santa Barbara, CA, USA.

A tunable pulsed mid–IR Quantum Cascade Laser (QCL, four chips) was used to create a small photothermal effect in the sample. The probe laser (green 532 nm) was used to detect and collect this rapid conversion of photon energy into heat.

The QCL was operated with 300 ns pulses at a repetition rate of up to 100 kHz, corresponding to a maximum duty cycle of 5%. The focused spot size of the green probe laser was about 500 nm to deliver submicron spatial resolution. Spectra were collected within the region of 1900–950 cm^−1^; the number of co-added scans per spectrum was optimised during the study and was equal to 8. The laser powers of the IR and probe were each set to approximately 1–5 mW per spectrum.

Prior to the experiments, optical images of the sample were recorded using Cassegrain objectives: low magnification 10 X, 0.3NA and high magnification 40 X, 0.78NA (with working distances of about 15 and 8 mm, respectively). The latter was also used for O-PTIR spectra collection, which were saved and processed in PTIR Studio 4.4.7986 software provided with the instrument. Subsequently, the instrument was wavenumber-calibrated using the test sample (cross-sections of PMMA and PS beads embedded in epoxy deposited on ZnS window; 200–800 nm thick). The effective spectral resolution was set to 6.6 cm^−1^. The sample was placed on the automated scanning stage in the sample chamber and purged with dried nitrogen during all experiments to eliminate water vapour absorption. Before each experimental session, the background signal was recorded (under the same experimental conditions) to consider the influence of the IR laser power distribution on the final spectrum.

For this study, we focused on collecting hyperspectral image data from different areas of the analysed sample. A hyperspectral image is defined in PTIR Studio as a raster scanning. A single spectrum is collected from one point, then the sample is moved, and the next spectrum is collected from the new location. The size of the single hyperspectral object was set to 19 × 13.5 µm^2^, whereby 1092 spectra were collected with a spacing of 0.5 µm in *x* and 0.5 µm in *y* directions (acquisition time around 5 h). This set of parameters ensured taking full advantage of the wavenumber-independent sub-micron spatial resolution of the O-PTIR technique.

For map creation, open-source ImageJ software was used. Numerical interpolation (bicubic) between the experimental points (pixels) was applied; this is the standard approach for both commercial and open-source software when low-resolution (small number of pixels in *x* and *y* direction) images are recorded and must be presented in graphical form. ATR–FTIR maps were 64 pixels × 64 pixels, whereas O-PTIR maps were 39 pixels × 28 pixels.

## 3. Results

### 3.1. Sample Description

The paint fragment consisted of different degraded compounds from zinc white oil paint. Previously, the surface haze developed on different parts of the painting had been analysed by transmission-FTIR microspectroscopy and were identified as zinc-type degradation products consisting of zinc soaps, oxalates, hydroxychlorides, sulphates, and carbonates, postulated to have formed at the paint’s surface from the natural ageing of oil binder and interaction with atmospheric pollution [8].

Figure 1b shows the cross-section with a whitish haze over an underlying blue paint containing Prussian blue. The corresponding SEM image shows, in detail, a heterogeneous mix of aggregate-like structures at the haze–paint interface. The upper haze is very thin, about 1–2 µm, and appears intimately bound to discrete soap aggregates (Figure 1c).

The characteristic FTIR absorbance bands for each zinc-type degradation compound are as follows:

- Zinc stearate/palmitates or zinc soaps; amorphous soaps are represented by a strong, broad band at 1590 cm^−1^, and crystalline soaps are represented by a sharp band at 1540 cm^−1^. Medium peaks of carboxylate stretching at 1465 and 1398 cm^−1^ could also be observed [9,10];
- Gordaite (NaZn_4_Cl(OH)_6_SO_4_·6H_2_O) shows characteristic sulphate stretching bands at 1120 cm^−1^ and 990 cm^−1^ (SO_4_^2−^ stretching modes), 1670 cm^−1^, and 1639 cm^−1^ (HOH bending), and three distinct peaks at 3347 cm^−1^, 3401 cm^−1^, and 3505 cm^−1^ (OH stretching in the metal hydroxide layer, or structurally bound water) [8,11,12];
- Zinc lactate (a water-soluble salt less commonly detected on paintings) is characterised by a strong, broad band at 1590 cm^−1^, accompanied by a series of distinct narrower bands at 1367, 1321, 1273, 1119, 1085, and 1046 cm^−1^ [13,14].

The experiment aimed to identify zinc-type degradation products in the cross-section with the ATR–FTIR and O-PTIR techniques by integrating the characteristic peak of each compound. Zinc soaps (amorphous and crystalline), gordaite, and zinc lactate were selected for comparison. Their characteristic peaks for integration were 1590 cm^−1^, 1540 cm^−1^, 1120 cm^−1^, and 1270 cm^−1^, respectively. Prussian blue was left out of the comparison study as its characteristic marker at 2090 cm^−1^ is out of the spectral range for O-PTIR (cutoff at 1900 cm^−1^). The spectral range chosen for comparison was between 1900 cm^−1^ and 950 cm^−1^ to incorporate both techniques.

### 3.2. ATR–FTIR Imaging Results

**Figure 2:**
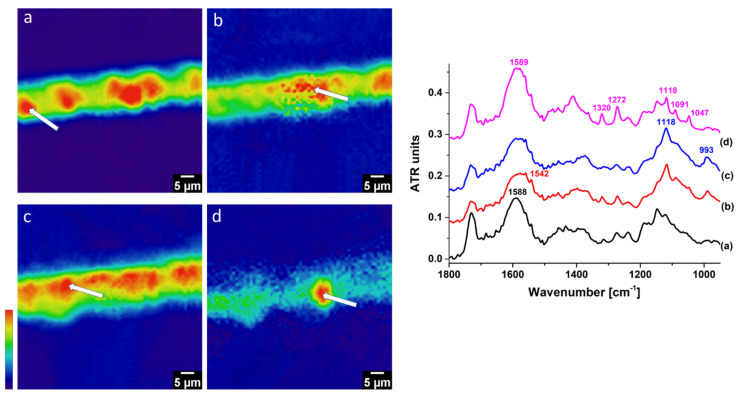
ATR–FTIR FPA imaging from the centre spot (70 × 70 µm^2^) and representative spectra: (**a**) amorphous zinc soap (1590 cm^−1^), (**b**) crystalline zinc soap (1540 cm^−1^), (**c**) gordaite (1120 cm^−1^), and (**d**) zinc lactate (1270 cm^−1^). Red denotes the highest intensity, and blue denotes the lowest intensity. For clarity of presentation, the experimental artefacts from the areas outside the sample were removed. ATR–FTIR spectra were taken from the spot with the highest intensity within the imaging representative for a selected wavenumber (marked by the white arrows).

At least three hyperspectral areas, each about a 70 × 70 µm^2^ area, were tested along the length of the cross-section. For comparison, results from the centre spot for ATR–FTIR imaging is shown in Figure 2.

Amorphous zinc soaps were detected throughout the cross-section, as shown by a strong, broad carboxylate band at 1590 cm^−1^ in all the spectra (Figure 2a). Positive identification of crystalline zinc soaps was less apparent, as seen by the weak, sharp band at 1540 cm^−1^. Due to the low signal-to-noise ratio for crystalline zinc soaps, the resulting FPA image was unclear and was not accurately representative (Figure 2b). It should be noted that the weak peak at 1540 cm^−1^ in crystalline zinc soaps, especially when present as tiny particles at sub-micron size, can easily go unnoticed. In other spots of the cross-section, it is possible to occasionally obtain a higher signal-to-noise at 1540 cm^−1^; presumably, this is because larger aggregates of crystalline soaps were available for detection.

Gordaite could not be well-resolved in the cross-section (Figure 2c), which was expected to form at the uppermost layer of the cross-section in the SEM image (Figure 1c). As seen in Figure 2, the FPA images for gordaite (Figure 2c) and amorphous zinc soaps (Figure 2a) overlapped in the same layer. The inability to spatially localise gordaite was also encountered in other tested spots of the cross-section, indicating that its size is below the detectable IR diffraction limit.

On the contrary, zinc lactate was well-resolved as an agglomerate highly concentrated at the centre (Figure 2d).

### 3.3. O-PTIR Imaging Results

**Figure 3:**
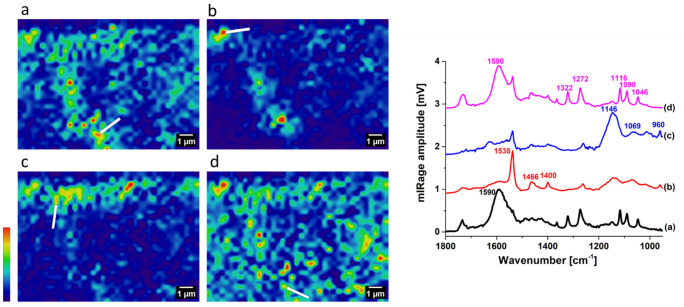
O-PTIR hyperspectral imaging from the centre spot (19 by 13.5 µm^2^) and representative spectra: (**a**) amorphous zinc soap (1590 cm^−1^), (**b**) crystalline zinc soap (1540 cm^−1^), (**c**) gordaite (1146 cm^−1^), and (**d**) zinc lactate (1270 cm^−1^). Red denotes the highest intensity, and purple denotes the lowest intensity. Characteristics for selected wavenumber O-PTIR spectra were taken from the spots marked by white arrows.

At least 10 hyperspectral areas were tested along the cross-section. For comparison, results from the centre spot for O-PTIR imaging (19 × 13.5 µm^2^) are shown in Figure 3.

The hyperspectral images obtained by O-PTIR appeared speckled, and gaps in the cross-section that were not at the same polished level showed up more readily due to lower signal. Amorphous zinc soaps can be identified in all spots with a broad carboxylate band at 1590 cm^−1^, and their occurrence was widespread throughout the cross-section (Figure 3a). Crystalline zinc soaps were readily detected as localised aggregates with a better signal-to-noise ratio, even though minute amounts were available in the centre spot, demonstrating the ability of O-PTIR to detect trace quantities (Figure 3b).

Gordaite could be well-resolved with O-PTIR, and its presence was highly concentrated in the upper part of the cross-section (Figure 3c). When the hyperspectral image for gordaite was overlaid with the SEM image, it showed comparable consistency (Figure 1c). The ability to resolve gordaite with O-PTIR was also affirmed in other tested areas of the cross-section. Interestingly, the characteristic peaks for gordaite in O-PTIR (1146, 960 cm^−1^) differed from those obtained with ATR–FTIR (1118, 1069, 993 cm^−1^). It is unlikely that this was due to a difference in measurement technique and could more likely be attributed to the variation in gordaite symmetry in the sample. The stretching bands of sulphate in gordaite have been reported to occur in a range of values by different authors [11,12]. The shift to a lower S–O stretching frequency at 960 cm^−1^ implies slight distortion of sulphate groups due to hydrogen bonding [12].

Zinc lactate was positively identified with excellent signal-to-noise (Figure 3d). Like amorphous zinc soaps, zinc lactate appeared widespread in the tested area. It should be mentioned that zinc lactate was not detected in other areas along the cross-section, and this result corresponded to that obtained by ATR–FTIR imaging (Figure 2a). With O-PTIR hyperspectral imaging, the thin layers and small components of interest could be visualised spatially at sub-micron level.

## 4. Discussion

### 4.1. Comparing O-PTIR and ATR–FTIR Results

**Figure 4:**
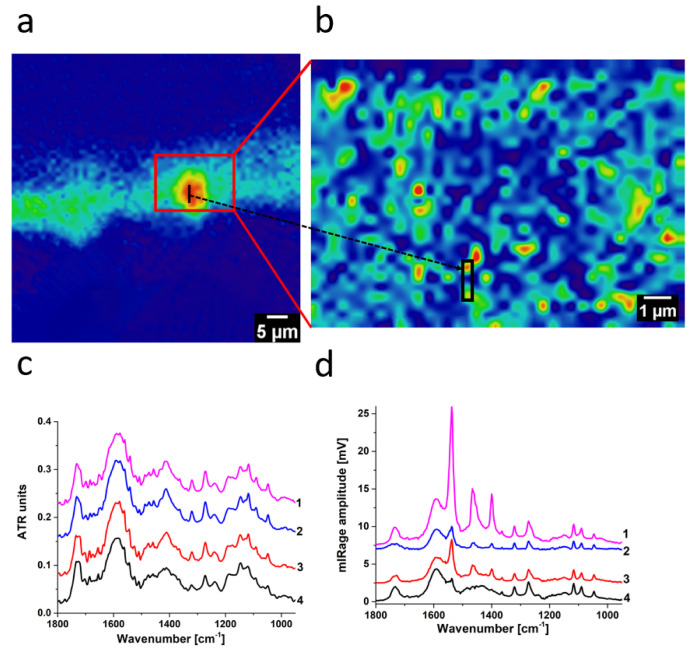
Comparison of the ATR–FTIR (FPA) and O-PTIR results: distribution of the integral calculated for 1270 cm^−1^ for zinc lactate collected for (**a**) ATR–FTIR and (**b**) O-PTIR. Red rectangle in (**a**) denotes the area in (**b**) analysed by O-PTIR spectroscopy. Four consecutive spectra are recorded from the points marked as (**a**) black line, (**b**) black rectangle. Distance between spectra is 1.1 µm for (**c**) ATR–FTIR and 0.5 µm for (**d**) O-PTIR.

To compare the two techniques, we selected four consecutive point spectra in an area containing zinc lactate (Figure 4). Major absorbance peaks in zinc lactate were virtually identical between O-PTIR and FTIR spectra, demonstrating that both techniques could be used to interpret transmission-like FTIR library spectra. Slight differences were visible and acceptable as FTIR spectroscopy depends not only on the samples’ infrared absorption characteristics, but also on refractive index, scattering, and dispersion artefacts.

Interestingly, two of the O-PTIR selected spectra showed an intense sharp band at 1540 cm^−1^ on top of the absorbance peaks of zinc lactate. This result indicated that crystalline zinc soaps occurred with zinc lactate in the two spots. On the other hand, ATR–FTIR showed consistent spectra in all four points, demonstrating that zinc lactate was the bulk component. Although it did not pick up any crystalline zinc soaps with zinc lactate, it does not mean that they were absent. Therefore, combining ATR–FTIR and O-PTIR imaging for the same sample is advantageous in presenting a more holistic picture of the sample information.

It is worth pointing out here that the hyperspectral approach of O-PTIR, discussed in this paper, is very similar to experiments performed by means of a conventional FTIR microscope with an FPA detector. However, in a conventional FTIR microscope, the pixel resolution depends on the type of objective mounted on the IR microscope. For the ATR objective in this work, the theoretical spatial resolution was within the range of 0.62–6.2 μm (0.25 λ) [15], which is highly wavenumber (wavelength)-dependent. Significantly, worse spatial resolution was obtained for low wavenumbers (higher wavelengths). This was in stark contrast to the O-PTIR technique, which provided constant, submicron spatial resolution across the entire IR spectrum. The spatial resolution in O-PTIR is defined by the wavelength and the spot size of the short wavelength green laser, not the long wavelength IR source.

### 4.2. Implications for Conservation

From the IR imaging results, it can be concluded that the upper layer of haze (i.e., gordaite) envelops the paint surface. Below the haze lies a mixture of zinc soaps, both amorphous and crystalline, infused with a zinc lactate aggregate.

The problems with oil paint containing zinc white have been well reported in a number of studies, whereby crystalline zinc soaps appear to play a key role in the loss of binding ability and, subsequently, paint delamination and flaking [16,17,18,19]. Accounts of crystalline zinc soaps concentrated at the surface of grounds and in contact with higher binder oil were mentioned. In this case, the crystalline zinc soaps appeared as discrete, localised aggregates in low levels held within an amorphous ionomeric network. This explains why the painting is still structurally stable and yet to arrive to the state of flaking. As the paint continues to age, it is expected that increasing crystallinity leads to a loss in structural integrity and eventually paint failure.

Zinc lactate is another degradation product of oil paint less commonly reported in the conservation literature, with only a few accounts mentioning its presence in paintings; it is postulated to be formed from an atmospheric reaction or lactic acid inherently within the painting [13,14,20]. The high water-solubility of zinc lactate implies that this painting is water-sensitive. Experimentally, this painting has exhibited paint loss when water swabs were applied in some areas and not others; however, the exact reasons for its water sensitivity are unclear [8]. Combining the results from ATR–FTIR and O-PTIR, it becomes clear that in some areas, zinc lactate is localised (Figure 2d) below the gordaite layer (Figure 3c). Even though gordaite is water-insoluble, hence in a way behaving as a barrier for the painting, water on the surface can penetrate surface paint cracks and leach out water-soluble zinc lactate and surrounding pigments, leading to paint loss. It can thus be deduced that areas where underlying zinc lactate agglomerates thrive, or areas with more surface cracks, are relatively more sensitive to surface cleaning.

## 5. Conclusions

O-PTIR is an emerging non-invasive spectroscopy technique in the heritage field. To the best of our knowledge, this is the first example that compares the state-of-the-art O-PTIR technique with the conventional ATR–FTIR imaging technique in analysing a cultural heritage sample. A key advantage proposed for the O-PTIR technique, and our primary interest for the comparative study, is its ability to accurately characterise and spatially resolve different compounds below the IR diffraction limit.

Presented in this paper is the characterisation of a cross-section embedding a thin paint fragment that has delaminated from the surface of a hazy oil painting. The zinc white oil paint is heavily degraded with natural formations of different compounds of gordaite, zinc lactate, and zinc soaps (amorphous and crystalline). Knowing the spatial locations of the degradation compounds characterised at the haze–paint interface is beneficial to understanding the painting’s condition for conservation.

ATR–FTIR showed that zinc lactate is localised as an agglomerate and not a continuous layer, while the rest of the cross-section contained zinc soaps of palmitates and stearates, mainly amorphous. Crystalline zinc soaps were not as readily detected, likely because very little was present or that the particles are too small. Although gordaite could be identified, the gordaite layer < 2 µm is too thin to be well-resolved. Placing the same sample into O-PTIR, gordaite is excellently resolved as a thin continuous layer at the upper portion of the cross-section, and this information complements the SEM image. Additionally, O-PTIR is able to detect trace levels of crystalline zinc soaps as localised aggregates with better signal-to-noise compared to ATR–FTIR. While ATR–FTIR presents a broad overview of the sample information, O-PTIR is beneficial in probing for tiny discrete particles or thin layers at sub-micron level.

The obtained experimental results suggest that an O-PTIR-based microscope could open a new way for the non-destructive, efficient, and reliable analysis of paint multilayers, especially those that contain thin layers or discrete components below the IR diffraction limit. O-PTIR’s excellent spectral quality, sub-micron spatial resolution, and high lateral resolution may help it to become an essential analytical tool for cultural heritage science.

## Data Availability

The data presented in this study are available on request from the corresponding author.
